# Deep Learning Plus Three-Dimensional Printing in the Management of Giant (>15 cm) Sporadic Renal Angiomyolipoma: An Initial Report

**DOI:** 10.3389/fonc.2021.724986

**Published:** 2021-11-15

**Authors:** Yunliang Gao, Yuanyuan Tang, Da Ren, Shunhua Cheng, Yinhuai Wang, Lu Yi, Shuang Peng

**Affiliations:** ^1^ Department of Urology, The Second Xiangya Hospital, Central South University, Changsha, China; ^2^ Department of Oncology, The Second Xiangya Hospital, Central South University, Changsha, China; ^3^ Hunan Engineering Research Center of Smart and Precise Medicine, Changsha, China; ^4^ Clinical Nursing Teaching and Research Section, The Second Xiangya Hospital, Central South University, Changsha, China

**Keywords:** deep learning, three-dimensional printing, giant, kidney, angiomyolipoma, partial nephrectomy

## Abstract

**Objective:**

To evaluate the feasibility and effectivity of deep learning (DL) plus three-dimensional (3D) printing in the management of giant sporadic renal angiomyolipoma (RAML).

**Methods:**

The medical records of patients with giant (>15 cm) RAML were retrospectively reviewed from January 2011 to December 2020. 3D visualized and printed kidney models were performed by DL algorithms and 3D printing technology, respectively. Patient demographics and intra- and postoperative outcomes were compared between those with 3D-assisted surgery (3D group) or routine ones (control group).

**Results:**

Among 372 sporadic RAML patients, 31 with giant ones were eligible for analysis. The median age was 40.6 (18–70) years old, and the median tumor size was 18.2 (15–28) cm. Seventeen of 31 (54.8%) had a surgical kidney removal. Overall, 11 underwent 3D-assisted surgeries and 20 underwent routine ones. A significant higher success rate of partial nephrectomy (PN) was noted in the 3D group (72.7% *vs*. 30.0%). Patients in the 3D group presented a lower reduction in renal function but experienced a longer operation time, a greater estimated blood loss, and a higher postoperative morbidity. Subgroup analysis was conducted between patients undergoing PN with or without 3D assistance. Despite no significant difference, patients with 3D-assisted PN had a slightly larger tumor size and higher nephrectomy score, possibly contributing to a relatively higher rate of complications. However, 3D-assisted PN lead to a shorter warm ischemia time and a lower renal function loss without significant difference. Another subgroup analysis between patients under 3D-assisted PN or 3D-assisted RN showed no statistically significant difference. However, the nearness of tumor to the second branch of renal artery was relatively shorter in 3D-assisted PN subgroup than that in 3D-assisted RN subgroup, and the difference between them was close to significant.

**Conclusions:**

3D visualized and printed kidney models appear to be additional tools to assist operational management and avoid a high rate of kidney removal for giant sporadic RAMLs.

## Introduction

Renal angiomyolipoma (RAML) is the most common solid benign tumor of the kidney, typically composed of dysmorphic blood vessels, smooth muscle, and mature adipose tissue with varying proportions (1). The estimated incidence of RAMLs in the general population is about 0.13%, predominately in women (2, 3). Approximately 80% RAMLs can occur sporadically or, less commonly, as part of tuberous sclerosis complex (4). The main clinical concern of RAMLs is the risk of life-threatening hemorrhage caused by spontaneous tumor rupture. With the increasing in tumor size, the risk of hemorrhage could increase correspondingly (4). Sporadic ones present a relative slow growth rate but could grow over 15 cm (referred to as “giant”) (5, 6). Despite marked advances in embolization or cryoablation, therapeutic algorithms for such giant RAMLs remain a considerable challenge. This is likely to be worse particularly in developing countries. Partial nephrectomy (PN) is a highly recommended treatment, but radical nephrectomy (RN) appears to be mostly applied due to operating difficulty and definitive outcomes (e.g., less perioperative complications, complete removal a suspected malignant tumor) (7–9). Therefore, how to facilitate PN procedure for these giant ones is worth to explore.

Three-dimensional (3D) printing is rapidly advancing in the management of different urological diseases, such as small renal cancer, adrenal cancer, and prostate cancer (10). With high fidelity to the original organs, 3D printing is capable of providing a deep knowledge of topographic anatomy, a description of spatial relationship, and a sense of touch when compared with traditional two-dimensional images (11). However, several concerns have limited its use in precise surgery, including absent quantified measurements, redundant data presentation, time dependence, and object deformation (12). Fortunately, with rising achievement of computer science in medicine, artificial intelligence (AI) with deep learning (DL) algorithm could offer a more precise and transversal view of a clinical scenario. DL represents the latest iteration in a progression of AI technologies. It has recently shown promising performance in various medical tasks, including image synthesis, disease diagnosis, and predictive analysis (13). Particularly, DL in the field of renal diseases enables to autonomically classify renal mass, differentiate tumor grades, evaluate acute kidney injury, and so on (14). DL is currently revolutionizing and reshaping previous medical care strategies.

Given the high rate of intraoperative kidney removal in giant sporadic RAML patients, novel approaches are imperative to maximize preservation of renal function. The advent of DL and 3D printing sheds light on this critical issue to meet surgeons’ and patients’ expectations. Thus far, to our knowledge, no study has demonstrated the superiority of DL and 3D printing application in surgical management over traditional ones for giant RAMLs. Therefore, we constructed 3D visualized and printed kidney models to assist PN for giant sporadic RAML patients, hoping to better understand individual tumors and improve surgical outcomes.

## Methods

### Study Population

A retrospective analysis was performed of all sporadic RAML subjects who received 3D models-assisted surgery (3D group) or routine one (control group) in our medical center between January 2011 and December 2020. Study approval was obtained from the Ethics Committee of the Second Xiangya Hospital (No. LYF2020102). All patients were fully informed about the advantages and disadvantages of the 3D visualized and printed kidney models (since these techniques introduced to our medical center), and the patients had their own right to determine whether to use. RAML was preoperatively diagnosed by ultrasonography and computed tomography (CT) and postoperatively confirmed by histopathology. Tumor size was defined as the greatest diameter recorded on pathology report or radiological imaging, with the priority of pathological report, MRI, CT, and echo. Based on this definition, patients with RAML ≥15 cm were enrolled for analysis. Those patients with tuberous sclerosis complex, suspicion of malignancy, or multiple bilateral lesions were excluded. All patients’ clinical characteristics were extracted from medical records for analysis. The RENAL nephrometry score (15) was applied to quantify tumor anatomical complexity for effective comparison.

### DL and 3D Segmentation

**Figure 1:**
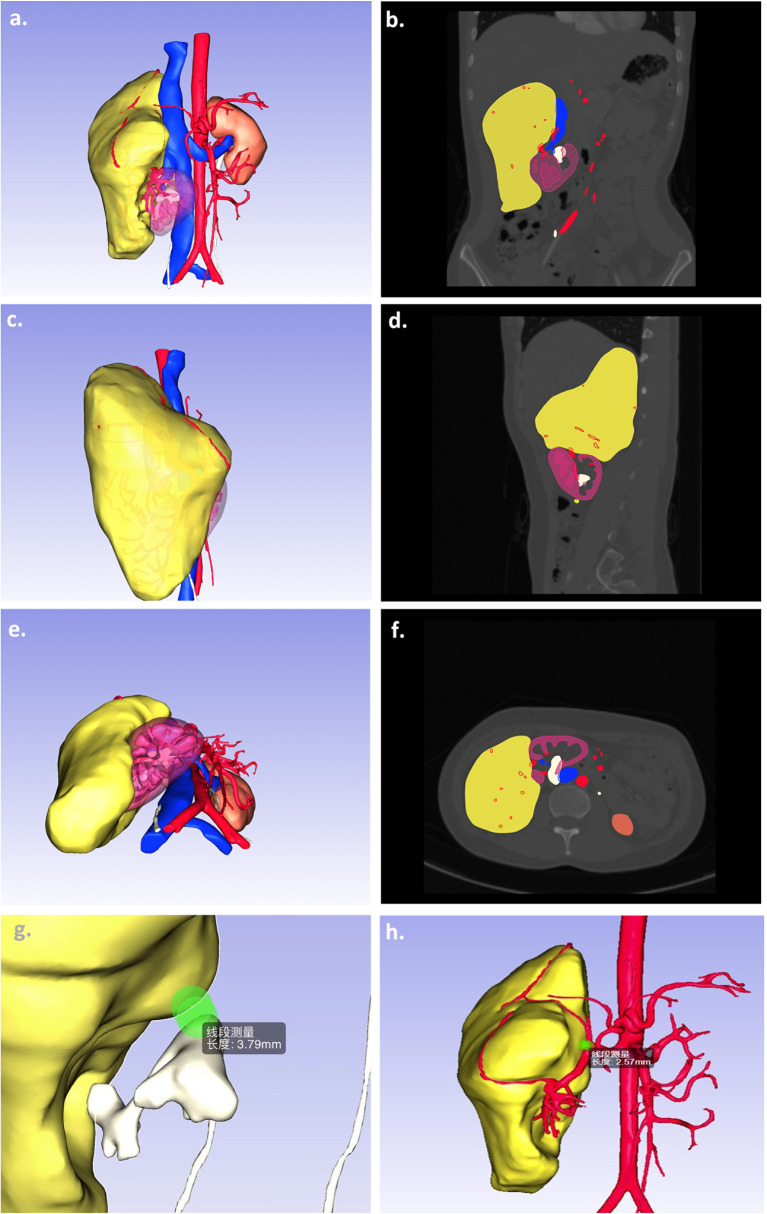
A sample of 3D segmentation of giant sporadic renal angiomyolipoma. Computed tomography scans of kidney tumors show the kidney (purple), tumor (yellow), arteries (red), veins (blue), and collecting system (white) segmentations as overlays **(A, C, E)**. The images **(B, D, F)** present the corresponding 3D segmentations at different view positions. The other images demonstrated the important parameters, namely, the nearness of the tumor to collecting system **(G)** and the nearness of tumor to the second branch of renal artery **(H)**.

Prior to surgery, all patients’ CT data (Siemens Corporation, Germany) were applied to image synthesis. DL method nnU-Net (16, 17) trained on KiTS19 (18) (with DICE 91%) and fine-tuned using our additional 30 patients was used to segment the kidney and the tumor. Vein, artery, and collecting tubular system were then segmented from the CT images using multiscale region growing method built in the commercial annotation tool (Hcit.ai Co., Ltd.). Meshes were then generated from the segmentations using marching cubes algorithm. Three important shortest distance between surfaces were then calculated for clinical comparison (named D.N.N): (D)epth of tumor into renal parenchyma, (N)earness of the tumor to collecting system, and (N)earness of tumor to the second branch of renal artery. Besides, the ratio of tumor volume/renal volume was determined. An example of segmented RAML imaging is presented in **Figure 1**. The image could provide a rendered view of an arbitrary view position and orientation (see **Supplementary Material 1**).

### 3D Printing

Individualized 3D printed physical kidney model was generated as previously described (19). All obtained CT data were imported to Mimics 19.0 system (Materialise, Belgium) for 3D reconstruction. After that, a 3D printer was employed to model and fabricate each kidney including the entire renal unit with the lesion *in situ*. The printed 3D model was made of photosensitive resin and mixed with colorant materials to represent the tumor, renal parenchyma, pelvis, vessels, and other structures.

### Procedure

The 3D visualized and printed physical models were mainly used for preoperative surgical planning and intraoperative navigation (e.g., mapping supply vessels). Prior to surgery, experienced surgeons would inspect tumor characteristics, arteriovenous distribution, collection system, and adjacent structures. Importantly, the accurate data about D.N.N and volume ratio were evaluated carefully for the feasibility and potential complications of PN. Patients in the 3D group were operated under the guidance of LY (the corresponding author with about 30 years’ experience). Patients in the control group received surgeries from LY and other two chief well-skilled surgeon, respectively.

All patients were treated through a transperitoneal open approach with a subcostal incision. The patient was placed in the supine position under general anesthesia. After exploring the abdominal cavity, the huge mass was observed obviously. The 3D models were explored intraoperatively to map the main supply vessels of both the kidney and the tumor. The renal hilum was dissected completely, and the major branches of the renal artery were clamped when needed. The renal vein was commonly left intact. The mass was meticulously isolated from the surrounding tissues, and the supply vessels were ligated. Based on 3D models and intraoperative findings, PN would be preferred; otherwise, RN would be performed. For those patients undergoing conventional surgery without 3D assistance (control group), the choice between PN and RN was also made according to preoperative imaging and intraoperative findings, with priority to PN over RN. The root of RAML was excised circumferentially from normal parenchymal by using a combination of blunt and sharp dissection. The collecting system was checked, and the capsular defect was reconstructed carefully. A drainage tube was placed after hemostasis. Those patients without 3D assistance were received routine PN/RN surgery.

### Outcome Measures and Statistical Analysis

A descriptive analysis of patient demographics was conducted, including age, gender, American Society of Anesthesiologists (ASA) status, tumor size, side of treated, history of prior selective arterial embolization (SAE), and RENAL score. Surgical details mainly included intraoperative results [operation time, warm ischemia time (WIT), estimated blood loss (EBL), intraoperative complications, intraoperative red blood cell (RBC) transfusion] and postoperative results (change in hemoglobin, change in renal function, postoperative complications, the ratio of kidney removal, the time of drainage tube removal, and hospital stay). Renal function was evaluated as estimated glomerular filtration rate (eGFR) by using the Chronic Kidney Disease Epidemiology Collaboration (CKD-EPI) equation (20). Postoperative hemoglobin and renal function were tested on the first day after surgery. The postoperative complications were assessed according to the Clavien–Dindo grading system (21). Long-term prognosis was not assessed due to the short follow-up period for some patients undergoing 3D-assitant surgery.

Data were presented as means ± standard deviation (SD) for normally distributed continuous values and as median with range for non-normally distributed data, while discrete ones were reported using proportions. Student’s t-test, chi-square test, and Mann–Whitney test were used for statistical analysis as appropriate. All available data were statistically analyzed using IBM SPSS Statistics 24.0 software package. A statistical difference was considered when a p < 0.05.

## Results

A total of 372 sporadic RAML patients were identified in our database. Among them, 31 (8.3%) with RAMLs over 15 cm were eligible for analysis. The median age was 40.6 ± 12.8 (range, 18–70) years old, and 28 (90.3%) were female. The median tumor size was 18.2 ± 3.2 (range, 15–28) cm, and the median RENAL score was 8.2 ± 1.5. Seventeen out of 31 (54.8%) patients had a surgical kidney removal. Overall, 11 underwent 3D-assisted surgeries (3D group), and the other 20 underwent routine ones (control group). **Table 1** presents a summary of patients’ characteristics. Two groups showed no significant differences in terms of age, gender, ASA status, tumor size, side of treated, the ratio of prior SAE, and RENAL score.

**Table 1:** Baseline demographic and clinical characteristics.

Patient Demographics	3D group (n = 11)	Control group (n = 20)	p-value
Age (years), mean ± SD (range)	38.9 ± 12.4 (23–64)	41.5 ± 13.2 (18–70)	>0.05
Gender (male/female), n	0/11	2/18	>0.05
ASA status (grade I/II/III), n	1/8/2	1/13/6	>0.05
Body weight (kg), mean ± SD (range)	56.6 ± 4.6 (51–65)	55.8 ± 10.0 (44–77)	>0.05
Tumor size (cm), mean ± SD (range)	18.0 ± 2.2 (15–22)	18.3 ± 3.6 (15–28)	>0.05
Left/right, n	6/5	11/9	>0.05
Prior SAE, n (%)	2(18.2%)	3(15%)*	>0.05
RENAL score, mean ± SD	7.9 ± 1.4	8.5 ± 1.7**	>0.05

ASA, American Society of Anesthesiologists; SAE, selective arterial embolization; SD, standard deviation.

*One patient received emergency selective arterial embolization prior to radical nephrectomy.

**Nine patients’ relevant data were unavailable.

**Table 2** displays four parameters based on DL segmented image. The mean of depth of tumor into renal parenchyma, nearness of the tumor to collecting system, and nearness of tumor to the second branch of renal artery was 16.2 ± 11.2 mm, 7.7 ± 4.1 mm, and 12.8 ± 8.7 mm, respectively. The ratio of tumor volume/renal volume was 688.5 ± 345.5%.

**Table 2:** Artificial intelligence analyzed parameters for surgical guidance.

Item	Mean	SD
Depth of tumor into renal parenchyma (mm)	16.2	11.2
Nearness of the tumor to collecting system (mm)	7.7	4.1
Nearness of tumor to the second branch of renal artery (mm)	12.8	8.7
Ratio of tumor volume/renal volume (%)	688.5	345.5

SD, standard deviation.

**Table 3** summarizes intra- and postoperative results for 3D and control groups. A significant higher success rate of PN was noted in the 3D group rather than in control (72.7% *vs*. 30.0%). Patients in the 3D group had a smaller change in eGFR (21.0 ± 32.8 *vs*. 25.6 ± 18.0 ml/min/1.73 m^2^) without statistically significant. However, patients in the 3D group experienced a relatively longer operative time (240.0 ± 78.6 *vs*. 194.1 ± 84.5 min), a greater EBL (654.5 ± 393.4 *vs*. 324.0 ± 299.0 ml), a larger postoperative change in hemoglobin (−28.1 ± 23.9 *vs*. −1.5 ± 17.3 g/L), and a higher postoperative morbidity (72.7% *vs*. 30.0%). Grade I/II/III/IV postoperative complications due to Clavien–Dindo in 3D and control groups were 2/7/0/1 and 4/2/1/0, respectively. No significant differences in the rate of intraoperative RBC transfusion, the time of drainage tube removal, and the length of hospital stay.

**Table 3:** Perioperative parameters and clinical outcomes.

Variable	3D group (n = 11)	Control group (n = 20)	p-value
Success rate of PN, n (%)	8 (72.7%)	6 (30.0%)	<0.05
Operation time (min), mean ± SD	240.0 ± 78.6	194.1 ± 84.5	>0.05
Estimated blood loss	654.5 ± 393.4	324.0 ± 299.0	<0.05
Intraoperative complications, n (%)	2 (18.2%)	0 (0.0%)	>0.05
Collecting system injury, n (%)	1 (9.1%)		
Pancreas injury, n (%)	1 (9.1%)		
Intraoperative RBC transfusion, n (%)	10 (90.9%)	14 (70%)	>0.05
Postoperative change in hemoglobin (g/L), mean ± SD	−28.1 ± 23.9	−1.5 ± 17.3*	<0.05
Postoperative change in eGFR (ml/min/1.73 m^2^), mean ± SD	21.0 ± 32.8	25.6 ± 18.0**	>0.05
Postoperative complications, n (%)***	8 (72.7%)	6 (30.0%)	<0.05
Postoperative RBC transfusion (grade 2)	7	2	
Emesis (grade 1)		1	
Fever (grade 1)	1	1	
Pain (grade 1)	1	2	
Hydrothorax (grade 3)		1	
Respiratory failure (grade 4)	1		
Time of drainage tube removal (days), mean ± SD	6.6 ± 2.4	6.6 ± 2.6	>0.05
Hospital stay (days), mean ± SD	15.2 ± 4.4	16.2 ± 5.6	>0.05

eGFR, estimated glomerular filtration rate; PN, partial nephrectomy; RBC, red blood cell; SD, standard deviation.

*Two patients’ relevant data were unavailable.

**Two patients’ relevant data were unavailable.

***Parts of patients where more than one type of complications occurred. Grade ranking was according to the Clavien–Dindo classification of surgical complications.

**Table 4** shows a subgroup analysis of eight and six PN patients in 3D and control groups, respectively. No statistically significant difference was found between these two subgroups, except for postoperative change in hemoglobin. However, patients with 3D-assisted PN had a slightly larger tumor (18.1 ± 2.3 *vs*. 16.9 ± 2.5 cm) and higher RENAL score (7.8 ± 1.5 *vs*. 6.7 ± 1.2), possibly contributing to a relatively higher rate of complications (75.0% *vs*. 33.3%). Instead, 3D-assisted PN lead to a shorter WIT (23.1 ± 10.6 *vs*. 27.5 ± 2.4 min) and a lower renal function loss (14.8 ± 25.97 *vs*. 19.1 ± 20.0 ml/min/1.73 m^2^) without significant difference.

**Table 4:** Subgroup analysis of perioperative parameters and clinical outcomes.

Variable	PN in 3D group (n = 8)	PN in Control group (n = 6)	p-value
Age (years), mean ± SD	39.3 ± 14.0	43.8 ± 15.1	>0.05
Gender (male/female), n	0/8	1/5	>0.05
Tumor size (cm), mean ± SD	18.1 ± 2.3	16.9 ± 2.5	>0.05
Left/right, n	4/4	3/3	>0.05
Prior SAE, n (%)	0(0%)	0(0%)	>0.05
RENAL score, mean ± SD	7.8 ± 1.5	6.7 ± 1.2*	>0.05
Operation time (minutes), mean ± SD	243.8 ± 91.7	202.5 ± 88.1	>0.05
WIT (min), mean ± SD	23.1 ± 10.6	27.5 ± 2.4	>0.05
Estimated blood loss	650.0 ± 414.0	375.0 ± 451.2	>0.05
Intraoperative complications, n (%)	2 (22.2%)	0 (0.0%)	>0.05
Collecting system injury, n (%)	1 (11.1%)		
Pancreas injury, n (%)	1 (11.1%)		
Intraoperative RBC transfusion, n (%)	8 (100.0%)	5 (83.3%)	>0.05
Postoperative change in hemoglobin (g/L), mean ± SD	−34.0 ± 21.8	−6.5 ± 16.2	<0.05
Postoperative change in eGFR (ml/min/1.73 m^2^), mean ± SD	14.8 ± 25.97	19.1 ± 20.0	>0.05
Postoperative complications, n (%)**	6 (75.0%)	2 (33.3%)	>0.05
Bleeding requiring RBC transfusion (grade 2)	5	1	
Pain (grade 1)	1	2	
Respiratory failure (grade 4)	1		
Time of drainage tube removal (days), mean ± SD	6.5 ± 2.7	7.8 ± 2.8	>0.05
Hospital stay (days), mean ± SD	14.8 ± 4.7	16.8 ± 3.7	>0.05

eGFR, estimated glomerular filtration rate; PN, partial nephrectomy; SAE, selective arterial embolization; RBC, red blood cell SD, standard deviation; WIT, warm ischemia time.

*Three patients’ relevant data were unavailable in PN group.

**Parts of patients where more than one type of complications occurred. Grade ranking was according to the Clavien–Dindo classification of surgical combinations.

**Table 5** indicates a subgroup analysis between patients under 3D-assisted PN or 3D-assisted RN. Patients in each subgroup showed no statistically significant difference in demographics, perioperative parameters, and clinical outcomes. However, patients with 3D-assisted RN had a slightly higher RENAL score without significant difference. Particularly, the nearness of tumor to the second branch of renal artery was relatively shorter in 3D-assisted PN subgroup than that in 3D-assisted RN subgroup (4.7 ± 4.0 *vs*. 15.8 ± 8.0 mm), and the difference between them was close to significant (p = 0.05).

**Table 5:** Subgroup analysis of perioperative parameters and clinical outcomes in 3D group.

Variable	PN in 3D group (n = 8)	RN in 3D group (n = 3)	p-value
Age (years), mean ± SD	39.3 ± 14.0	38.0 ± 9.5	>0.05
Gender (male/female), n	0/8	0/3	>0.05
Tumor size (cm), mean ± SD	18.1 ± 2.3	17.7 ± 2.0	>0.05
Left/right, n	4/4	2/1	>0.05
Prior SAE, n (%)	0 (0%)	2 (66.7%)	>0.05
RENAL score, mean ± SD	7.8 ± 1.5	8.3 ± 1.2	>0.05
Ratio of tumor volume/renal volume (%), mean ± SD	705.9 ± 386.1	642.2 ± 265.7	>0.05
Nearness of the tumor to collecting system (mm), mean ± SD	8.9 ± 4.3	4.7 ± 1.2	>0.05
Nearness of tumor to the second branch of renal artery (mm), mean ± SD	15.8 ± 8.0	4.7 ± 4.0	=0.05
Depth of tumor into renal parenchyma (mm), mean ± SD	17.0 ± 13.0	14.0 ± 4.9	>0.05
Operation time (min), mean ± SD	243.8 ± 91.7	230 ± 36.1	>0.05
Estimated blood loss	650.0 ± 414.0	666.7 ± 416.3	>0.05
Intraoperative complications, n (%)	2 (22.2%)	0 (0%)	>0.05
Collecting system injury, n (%)	1 (11.1%)		
Pancreas injury, n (%)	1 (11.1%)		
Intraoperative RBC transfusion, n (%)	8 (100.0%)	2 (66.7%)	>0.05
Postoperative change in hemoglobin (g/L), mean ± SD	−34.0 ± 21.8	−12.3 ± 26.1	>0.05
Postoperative change in eGFR (ml/min/1.73 m^2^), mean ± SD	14.8 ± 25.97	37.8 ± 49.4	>0.05
Postoperative complications, n (%)*	6 (75.0%)	2 (66.7%)	>0.05
Bleeding requiring RBC transfusion (grade 2)	5	2	
Fever (grade 1)		1	
Pain (grade 1)	1		
Respiratory failure (grade 4)	1		
Time of drainage tube removal (days), mean ± SD	6.5 ± 2.7	7.0 ± 2.0	>0.05
Hospital stay (days), mean ± SD	14.8 ± 4.7	16.3 ± 4.2	>0.05

eGFR, estimated glomerular filtration rate; PN, partial nephrectomy; SAE, selective arterial embolization; RBC, red blood cell SD, standard deviation.

*Parts of patients where more than one type of complications occurred. Grade ranking was according to the Clavien–Dindo classification of surgical complications.

## Discussion

Our study presented one of the largest pooled analyses of giant (>15 cm) sporadic RAMLs and first highlighted the feasibility of the combination of DL and 3D printing techniques in the management of giant ones. Compared with traditional approaches, 3D visualized plus printed models could provide more detailed and precise information to facilitate surgical procedures and improve better outcomes. As shown by our results, a significant higher success rate of PN was made for giant RAMLs with the assistance of 3D models.

Sporadic RAMLs possess different clinical presentations and treatments in comparison to tuberous sclerosis ones (4). They commonly occur in middle age (40 years old) women (2, 3), as supported by our present findings. Sporadic ones commonly grow slowly, and those over 15 cm (named as “giant”) are infrequent (22). In our results, however, up to 8.3% of surgical cases could grow over 15 cm. Despite the benign nature, RAMLs still own the potential to cause life-threatening hemorrhage (23, 24). The risk of hemorrhage could increase with larger tumor size (4). Giant sporadic RAML has its own unique features as follows. Due to the extremely large size and volume, it could block the exposure of the tumor root and renal vessels, leave less space for operation, and cause severe perirenal adhesion and easy-touch bleeding (fragility) (2). A large incision is frequently necessitated to extract such huge tumor. Additionally, a RAML may exophytically grow in the retroperitoneum and closely mimic a liposarcoma due to the high fat content on a radiological image (25). These multiple factors challenge the optimum route for giant RAML treatments.

Currently, only a scarcity of studies documented the treatment outcomes in giant RAMLs (7–9). Giant ones are generally given individualized therapeutic algorithm like PN, RN, SAE, and ablation (1). Minimal invasive approaches are commonly the top priority for consideration, while several issues have limited their use. For instance, SAE appears to be an efficacious choice, but about 30% patients need secondary embolization, obviously increasing the medical burden (1, 4, 26). Ablation is optional but not always available in many medical settings, particularly in developing countries (1). Inadequate experience for giant RAMLs may contribute to the uncertainty around the effectiveness and value of these minimal interventions. Moreover, complete removal of tumor burden is commonly considered by patients. Therefore, PN is often recommended for larger RAMLs, but most treated ones have been reported below 10 cm according to two recent reviews (1, 2). RN is finally adopted due to the considerable challenges, rendering a higher rate of surgical kidney removal (27–32). It could be supported by our data that intraoperative kidney removal was performed in 54.8% of all giant RAML patients. This situation was worse in patients who underwent routine ones without 3D assistance. Of note, PN for giant ones appears to develop more complications, in particular bleeding risk. Intraoperative bleeding was commonly seen, and 24 of 31 (77.4%) patients required RBC transfusion. Postoperative RBC transfusion was also the most frequent complications in all 31 patients. In our study, 5 of 31patients performed a prior SAE. Based on our experience, SAE partly aided in intraoperative hemorrhage control but was limited to minimize the tumor volume. Thus, a novel approach is imperative to improve the rate of kidney salvage and facilitate the technically challenging PN for giant RAML management.

Accumulating evidence has investigated the application of 3D printing technology to guide PN for renal mass treatment (10). Surgeons could greatly benefit from the 3D mold with a better knowledge of topographic anatomy and the spatial structure. However, 3D printing-assisted PN appears to be mostly implemented for renal tumor below 10 cm (19, 33). Up till now, there is yet no group specially using 3D printing for RAMLs management, especially those giant ones. Moreover, there still remains some limitations in 3D printing, such as imprecise measurement and redundant data presentation of targets (12). To overcome the limitations of 3D printing and obtain better outcomes, we first employed DL for the surgical treatment of giant RAMLs. As a subset of AI, DL is a data-driven algorithmic approach to mimic human intelligence in increasingly independent and sophisticated ways (13). At present, medical image analysis is the most successful applications of this burgeoning science in medicine. In terms of renal diseases, DL enables to autonomically classify renal mass, differentiate tumor grades, evaluate acute kidney injury, and so on (14). Three main DL methods are commonly applied for medical imaging synthesis including auto‐encoder, generative adversarial network, and U‐net. The U-net method is the most adopted one (18). In this work, we applied a novel path called nnU-Net to extract subtle information from CT images and facilitate surgical procedures. Developed by Isensee et al., nnU-Net is an autosegmentation framework with removal of manual steps in data processing and network engineering (16, 17). DL-based 3D segmentation is capable of quantitatively characterizing RAMLs and their host kidneys. As mentioned in *Methods*, we calculated four parameters of interest, including the volume ratio and three shortest distances named D.N.N: (1) the volume ratio is mainly to evaluate the value of PN surgery; (2) the (D)epth of tumor into renal parenchyma is to decide the depth for dissection; (3) the (N)earness of the tumor to collecting system is to assess the risk of perforation to collecting system; and (4) the (N)earness of tumor to the second branch of renal artery is to guide artery clamping and avoid artery damage.

With the help of combination of DL-based 3D segmentation and 3D printed mold, PN appears to be easier to perform than before, leading to a higher success rate of PN for giant RAMLs. In our study, 72.7% patients in 3D group successfully underwent PN surgery, which was only 30% in control group. Yet, patients in the 3D group experienced a slightly longer operative time and a higher postoperative morbidity, especially the blood transfusion requirement. A plausible explanation is that more PN surgeries have performed in 3D group, and the resection of such giant tumor is highly complicated and challengeable. Subgroup analysis (**Table 4**) indicated a relatively shorter WIT and a higher eGFR preservation rate in patients undergoing 3D-assisted PN when compared with those undergoing routine PN. However, patients with 3D-assisted PN had a slightly larger tumor and higher RENAL score, possibly contributing to a relatively higher rate of complications. As mentioned above, giant RAMLs often adhere to surrounding structures and cause uncontrolled bleeding. A larger surgical field is commonly needed to dissect the tumor, leading to an increased risk of perioperative bleeding. Therefore, these factors could contribute to a longer operation time and more perioperative complications. It was consistent with the study of Patard et al., which revealed that intra- and postoperative PN morbidity were significantly increased with larger tumor size (34). However, the high rate of postoperative morbidity appeared to be acceptable because the most one was just requiring short-term blood transfusion. Moreover, another subgroup analysis (**Table 5**) demonstrated that 3D-assisted PN unfortunately failed in three patients. The reasons for the failure of PN in 3D group appeared to be multifactorial, possibly due in part to the intraoperative difficulty of PN. RAMLs in patients with failed 3D-assisted PN owned a relatively higher RENAL score and were relatively closer to the collecting system and to the second branch of renal artery, respectively. Despite this fact, the success rate of PN became higher under the help of 3D models. Additionally, 3D visualized and printed models could aid preoperative counseling to help patients understand about their disease, surgical plan, and risks. In this scenario, patients appear to be more willing to accept PN surgery instead of RN. The confidence may be also enhanced to surgeons to perform PN. Taken together, the combination of DL and 3D printing could serve as an extra tool to improve surgical outcomes in giant RAML management.

Notably, 3D visualization techniques hold a promising future in the surgical field of urology. They have several advantages over the current 3D printing techniques, such as waiving the cost of time and fee to print physical models. They could provide more precise information of anatomical structure in the operative area and reliably guide preoperative plan design. Consistent with our present study, some other 3D visualization techniques have recently been introduced to the field of urology and achieved impressive outcomes. For instance, Porpiglia et al. presented an innovative approach for preoperative planning to facilitate PN for complex tumors by adopting 3D augmented reality (3D-AR) systems (35). This 3D-AR approach could improve the percentage of cases with selective clamping of second-order arteries and the rate of tumor enucleation, contributing to the technical refinement of robotic-assisted PN. Wake and colleagues also reported similar 3D-AR methods to provide preoperative guidance for robotic PN (36). Additionally, Wang et al. previously applied 3D visualization technology in laparoscopic PN to achieve an accurate visible image-guided tumor resection with ideal renal function preservation (37). Therefore, the state-of-the-art 3D visualization methods open promising avenues to facilitate surgery procedures for complex renal tumors.

Despite the strengths of this study, certain limitations should be addressed, including the retrospective nature, the limited size of the total population, and some parts of missing data. We only recruited 31 giant RAMLs out of 372 patients for analysis, partly reducing the confidence power. Additionally, we just evaluated perioperative hospital complications for a short follow-up period. Surgical outcomes, particular in controls, were based on several well-skilled surgeons during a period of time. This might affect surgical morbidity and the success rate of PN. Given the limited number of patients, we did not take this variable into account. Moreover, we estimated the feasibility and effectiveness by combing DL-based segmentation with 3D printing. How the superior performance of solo DL segmentation remains unclear and needs to be explored further. The consistency between DL-based segmentation and CT images was not conducted. A measuring error may exist when measuring the depth of tumor into renal parenchyma, partly due to the miss of actual margin of tumor-affected kidney. However, our present study is one of the largest pooled analyses of giant RAMLs and first introduces the combination of DL and 3D printing for renal tumor surgery. All the patients selected presented with >15 cm RAML, which may add value to the relatively small sample size. It provides valuable information that can be used when discussing treatment options for patients with giant RAML.

## Conclusions

This study explored the feasibility of the combination of DL and 3D printing techniques in the surgical management of RAMLs, particularly the giant ones. Our initial experience indicated that the combination of these two techniques presented promising potential for preoperative surgical planning and intraoperative navigation. More detailed and precise information could be provided to facilitate surgical procedures and improve surgical outcomes. It may therefore play a part in an ongoing progress in the clinical practice towards the integration of these novel techniques in the surgical routine for various complex renal tumors, not limited to giant RAMLs. Further studies are needed to delineate the effectiveness and efficiency of this combined method.

## Data Availability Statement

The original contributions presented in the study are included in the article/**Supplementary Material**. Further inquiries can be directed to the corresponding authors.

## Ethics Statement

The studies involving human participants were reviewed and approved by the Ethics Committee of the Second Xiangya Hospital. The patients/participants provided their written informed consent to participate in this study.

## Author Contributions

Study concepts: YG. Study design: YG and LY. Data acquisition: YG, DR, and SC. Quality control of data and algorithms: YG, YT, and YW. Data analysis and interpretation: YG, DR, and SP. Statistical analysis: YG and SP. Manuscript preparation: YG. Manuscript editing: YT, SC, LY, and SP. Manuscript review: YT, SC, LY, and SP. All authors contributed to the article and approved the submitted version.

## Funding

This study was supported by grants from the Health Commission of Hunan Province to LY (20200816) and to SC (C2019162), respectively; by the grant from National Natural Science Fonundatioin of China (81800669) to YG.

## Conflict of Interest

The authors declare that the research was conducted in the absence of any commercial or financial relationships that could be construed as a potential conflict of interest.

## Publisher’s Note

All claims expressed in this article are solely those of the authors and do not necessarily represent those of their affiliated organizations, or those of the publisher, the editors and the reviewers. Any product that may be evaluated in this article, or claim that may be made by its manufacturer, is not guaranteed or endorsed by the publisher.
